# Aggression and Harm-Avoidant Trait Impede Recovery From Internet Gaming Disorder

**DOI:** 10.3389/fpsyt.2018.00263

**Published:** 2018-06-26

**Authors:** Seung-Yup Lee, Hae Kook Lee, Soo-young Bang, Hyunsuk Jeong, Hyeon Woo Yim, Yong-Sil Kweon

**Affiliations:** ^1^Department of Psychiatry, Uijeongbu St. Mary's Hospital, College of Medicine, The Catholic University of Korea, Seoul, South Korea; ^2^Department of Psychiatry, Eulji University Eulji Hospital, College of Medicine, Eulji University, Seoul, South Korea; ^3^Department of Preventive Medicine, College of Medicine, The Catholic University of Korea, Seoul, South Korea

**Keywords:** gaming disorder, hostility, harm avoidance, recovery, prognosis, course, risk-taking

## Abstract

**Background:** Relatively little is known about which neuropsychological factors promote recovery from Internet gaming disorder (IGD).

**Methods:** With informed consents, a cohort study was conducted in Seoul metropolitan area, South Korea, to investigate the course of IGD in youths. At baseline, we assessed psychosocial measures and gaming related measures such as Young's Internet Addiction Test (IAT) and the Aggression Questionnaire. The Balloon Analog Risk Task was also performed to study risk-taking behavior. A total of 60 subjects demonstrating three or greater criteria in the diagnostic interviews on IGD and the IAT score of 50 or above were included. After brief parental coaching at baseline, the participants were followed up at 3 and 6 months (*n* = 31). The baseline characteristics were compared between the non-improved group (<10% improvement in IAT score) and the improved group (≥30% improvement in IAT score) using Mann-Whitney *U*-test or chi-squared tests with a two-tailed statistical significance of 0.05.

**Results:** The non-improved group and the improved group did not demonstrate significant differences regarding demographics or the IAT scores at baseline. However, the IAT scores were significantly higher in the non-improved group at both 3 and 6 months. The non-improved group was also more likely to display higher aggression and harm avoidance than the improved group at baseline.

**Discussion:** Youths with excessive gaming problems should be evaluated for aggression and harm avoidance since they contributed to a worse prognosis. For those with high aggression or harm avoidance, more active therapeutic interventions should be considered.

## Introduction

With the release of the 11th International Classification of Diseases draft, internet gaming disorder (IGD) is anticipated to become the second formalized addictive behavior disorder after gambling disorder.

However, most of the previous studies were cross-sectional with only 13 longitudinal studies reported in a recent systemic review (1). Moreover, there is a major limitation among them since all of these studies relied on self-reports to evaluate IGD. In addition, none provided answers to the important question of how the clinical course would be in patients diagnosed with IGD.

To the best of our knowledge, the current study is the first study to investigate the course of IGD established by clinical assessments. In addition to IGD measures, various psychological and neurocognitive measurements were administered to explore factors that promote or undermine recovery from IGD. The identification of hindering factors for IGD recovery will inform us as to which patients may require more clinical resources or attention to facilitate recovery.

## Methods

### Procedures

This is a multi-center study that shares many designs with the Internet user Cohort for Unbiased Recognition of gaming disorder in Early adolescence (iCURE) study conducted at schools (2). While the iCURE was aimed at studying the natural course of IGD, this study intends to investigate the clinical course of the IGD affected subjects.

All recruitment was done in three university hospitals in Seoul and Uijeongbu, South Korea. Patients who visited our addiction or child-adolescent clinics either voluntarily, by referral from the local community mental health services, or by referral from the school iCURE research team were eligible for inclusion.

Participants were assessed at baseline, 3, 6, and 12 months, respectively, with the final examination being optional. At baseline, a 15–20 min of brief parents/guardians coaching session was provided with a pocket parental guidance of 12 written pages. This study was reviewed and approved by the institutional review board of Uijeongbu St. Mary's Hospital, the Catholic University of Korea (UC15ONMI0072).

### Participants

From August 2015 to January 2018, a total of 130 participants were recruited. Prior to participation, written informed consent was attained from both the patients and their parents/guardians. To ensure the severity of the gaming problem, we included 60 subjects, who demonstrated three or greater positive criteria in the diagnostic interviews on IGD and a baseline Internet Addiction Test (IAT) score of 50 or above. Thirty-one subjects who remained as participants in the study at the 6-month follow-up mark were finally included.

### Measurements

#### Diagnostic interviews

The participants underwent face-to-face interviews with clinical psychologists using the Diagnostic and Statistical Manual of Mental Disorders, Fifth Edition (DSM-5) IGD criteria in addition to the symptom of “craving.” The nine DSM-5 IGD criteria were assessed as suggested by the DSM-5 working group (3). “Craving,” an important addiction symptom but not addressed by the DSM-5 was also assessed by verifying whether the interviewee experienced “strong urges to game” or “difficulties with suppressing gaming desires.”

The psychiatric comorbidity was also assessed by the Korean Kiddie-Schedule for Affective Disorders and Schizophrenia for School-Age Children (4).

#### Neuropsychological assessments

Baseline intelligence was measured using the Korean Wechsler Intelligence Scale for Children for participants under 16 years of age and the Korean Wechsler Adult Intelligence Scale for those aged 16 years or older. In addition, the four main components of verbal comprehension index, perceptual reasoning index, working memory index, and processing speed index were also scored.

The Balloon Analog Risk Task (BART) was completed using the E-prime software (Psychology Software Tools, Sharpsburg, PA, USA). A left-side button was pressed to inflate the balloon as many times as the participants wished before reaching an explosion point to earn 10 scores for each unexploded ballooning attempt. Before the explosion, the participants could press a right-side button to stop the ongoing task and save the scores before losing by an explosion. After instruction, they performed 10 trials prior to the real experiment of 100 BART. The total saved scores and BART index were recorded. The BART index indicates the average frequency for ballooning in the unexploded trials. A high BART index indicates a stronger risk-taking trait while a low BART index indicates a higher harm avoidance trait (5).

#### Self-measurements

This study shares the majority of its study design with the iCURE study in regard to the self-measurements. For details, readers are advised to read the published protocol (2). For conciseness, the list and the internal consistencies of self-measurements will be provided as followings. The IAT (Cronbach α = 0.889) (6), the Korean Scale for Internet Addiction (Cronbach α = 0.96) (7), and the Short version of the Smartphone Addiction Scale (SAS-S) (Cronbach α = 0.967) (8) were used to assess problems related to digital media usage. In addition, psychological characteristics were measured using Barratt Impulsiveness Scale-11 (Cronbach α = 0.79–0.83) (9), the Attention Deficit Hyperactivity Disorder Rating Scale (ARS) (Cronbach α = 0.82–0.89) (10), the Aggression Questionnaire (Cronbach α = 0.889) (11), and the Rosenberg Self-Esteem Scale (Cronbach α = 0.75–0.87) (12).

### Statistical analyses

The inter-group comparison was performed according to the IGD recovery status after stratifying the included subjects according to the change in IAT score from baseline. At 6 months, those with an equal or greater than 30% reduction in IAT score from the baseline were defined as the improved group (*n* = 10). In contrast, those with worsening or a less than 10% improvement in IAT were classified as the non-improved group (*n* = 11). To identify factors that influence IGD recovery, the baseline characteristics were compared between the two groups. The Mann–Whitney *U* test and chi-squared/Fisher's exact tests were performed for continuous variables and categorical variables, respectively. Analyses were done using the SPSS Statistics for Windows, version 18 (SPSS Inc., Chicago, IL, USA) with a two-tailed statistical significance of 0.05.

## Results

No significant differences were found between the improved group and the non-improved groups in terms of the baseline demographics, digital media use measurements, or co-morbid psychiatric disorders (Table 1).

**Table 1:** Comparison between the non-improved and the improved groups at baseline.

	**Non-improved group (*n* = 11)**	**Improved group (*n* = 10)**	***p***
Age (years)	13.8 ± 3.0	12.2 ± 2.2	0.152
Male^†^	9 (81.8%)	8 (80%)	>0.999
Female^†^	2 (18.2%)	2 (20%)	>0.999
**DIGITAL MEDIA USE MEASUREMENTS**			
Number of positive IGD criteria^*^	5.1 ± 1.7	5.1 ± 1.7	0.973
Internet Addiction Test score	59.7 ± 5.6	68.6 ± 15.3	0.173
Korean Scale for Internet Addiction score	89.6 ± 13.7	82.5 ± 25.2	0.605
Smartphone Addiction Scale-Short score	35.6 ± 11.4	34.0 ± 12.9	0.918
**PSYCHIATRIC COMORBIDITIES**^†^			
Depression (+)	2 (18.2%)	2 (20%)	>0.999
Attention deficit hyperactivity disorder (+)	3 (27.3%)	4 (40%)	0.659
**PSYCHOLOGICAL MEASUREMENTS**			
Rosenberg Self-Esteem Scale	26.6 ± 5.5	28.7 ± 6.2	0.468
Barratt Impulsiveness Scale-II	62.6 ± 8.6	54.4 ± 5.3	0.085
Aggression Questionnaire	79.6 ± 16.8	61.3 ± 11.5	0.010
ARS	14.8 ± 10.1	24.6 ± 11.6	0.043
**NEUROCOGNITIVE ASSESSMENTS**			
BART index	27.1 ± 9.2	37.1 ± 9.3	0.024
Full Scale Intelligent Quotient	99.6 ± 18.7	88.1 ± 11.8	0.089
Verbal Comprehension Index	99.2 ± 16.6	98.8 ± 11.8	1.000
Perceptual Reasoning Index	108.4 ± 20.0	91.1 ± 14.1	0.043
Working Memory Index	98.6 ± 16.5	87.6 ± 13.8	0.123
Processing Speed Index	90.4 ± 13.2	83.8 ± 15.1	0.353

† *Fisher's exact test*;

* *Nine IGD DSM-5 criteria plus “craving.” IGD, Internet gaming disorder; ARS, Attention Deficit Hyperactivity Disorder Rating Scale; BART: Balloon Analogue Risk Task*.

However, the non-improved gamers demonstrated significantly higher aggression (79.6 ± 16.8 vs. 61.3 ± 11.5, *p* = 0.010). They also displayed lower BART index values (27.1 ± 9.2 vs. 37.1 ± 9.3, *p* = 0.024).

In the improved gamers, the ARS score (24.6 ± 11.6 vs. 14.8 ± 10.1, *p* = 0.043) was significantly higher and the perceptual reasoning index score (91.1 ± 14.1 vs. 108.4 ± 20.0, *p* = 0.043) was significantly lower when compared with the non-improved group.

**Figure 1:**
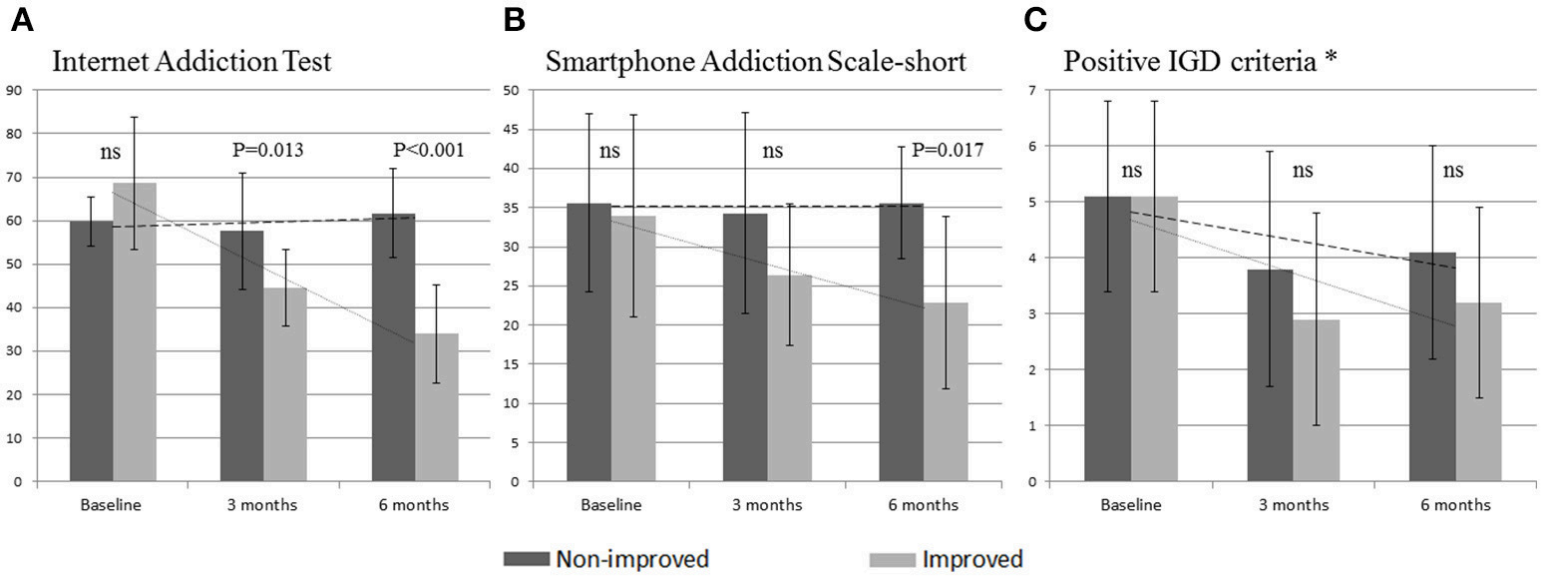
Longitudinal comparison of digital media use measures between the non-improved and the improved groups. **(A)** Changes of Internet Addiction Test scores. **(B)** Changes of Smartphone Addiction Scale-short. **(C)** Changes in number of positive IGD symptoms. ^*^Nine IGD DSM-5 criteria plus “craving”; IGD, Internet gaming disorder; ns, not significant; bar in the middle indicates SD.

Although there were no significant differences between the two groups at baseline, the IAT scores of the improved group were significantly lower at both 3 and 6 months than were the non-improved group's IAT scores. For SAS-S score, the difference became significant at 6 months, with lower scores demonstrated by the improved group. Although not significant, the number of positive IGD symptoms on the clinical interview also showed a tendency of improvement in the improvement group (Figure 1).

## Discussion

This study is the first to demonstrate the potential value of aggression and harm avoidance in the prognosis of IGD. Aggression has been repeatedly reported as a risk factor for IGD (13–15). High harm avoidance has been suggested as a vulnerable characteristic of IGD or substance use disorders (16, 17). The first strength of this study lies in its longitudinal design that enables inference on causality. Gamers with such risk factors were more likely to continue their gaming problems and to have a lower chance of self-recovery.

Gamers may regard online gaming platforms as an environment that meets their demand to release aggression or to reduce tension without inflicting adversary reactions from others. In numerous cross-sectional studies, aggression displayed an association with IGD (13–15). Aggression may also impede IGD recovery due to a failure in giving up the self-serving role of gaming to release their hostility. Moreover, gamers with high aggression could also be less compliant with parental guidance attempts to address their gaming behaviors. Further studies are required to reveal the mechanism of aggression as a poor IGD prognostic factor.

In typologies of alcohol use disorder and gambling disorder, the antisocial/impulsive type showed higher addiction severity (18–21). Recently, a theoretical IGD typology was suggested as “aggressive/impulsive,” “emotionally vulnerable,” and “socially conditioned” IGD subtypes (22). Our findings indicate that the IGD group with high aggression that corresponds to the antisocial/impulsive type in other addictive disorders may also have a worse prognosis.

To gamers with a high harm-avoidant trait, the virtual environment created by gaming may act as “a place to find peace.” They may regard virtual interpersonal interactions as relatively harmless compared to those that are in person in their daily lives. The secureness provided by gaming platforms in the context of social interactions may interact with the high harm-avoidant trait and in turn, undermines recovery from IGD. Therefore, screening and early interventions for high harm avoidance may improve the IGD treatment outcome.

The second major strength of this study is our effort to control parental influence. Parents may exert a significant influence on their children's gaming behavior, especially when their bond is strong and they communicate effectively. The IGD of children was negatively associated with autonomic and accepting parenting styles or the participation in enhanced social activities with their parents (23, 24). However, the majority of children reported rare or no supervision by their parents on their computer use duration and this lack of rule was reported as a risk factor for excessive use (25).

In addition to the strength of parent-child relationships, the effectiveness of parental guidance may vary widely according to the difference in parental knowledge on gaming issues. IGD is yet to be formalized in the medical system. Thus, awareness about the addictive potentials of this particular behavior may vary across households. Moreover, parents may not seek professional treatment immediately due to excessive concerns about labeling their children with an addiction stigma despite being aware of the potential harms.

Therefore, providing accurate information on IGD and parental coaching may decrease the gaps in IGD knowledge between households. We tried to minimize such variations by providing clear and uniform instruction to the parents. The parental coaching provided in this study included brief instruction on communication skills, setting rules, monitoring, and providing positive feedback to their offspring. However, no interventions were provided to the participants *per se* to better observe the clinical course of IGD.

However, there are also a number of limitations in this study. First, the sample size is relatively small and the possibility of selection bias cannot be ruled out. In addition to the small initial enrollment, only 31 subjects remained in the study at 6 months and contributed to the smaller final sample. However, our secondary analysis of baseline characteristics between the drop-outs and the final participants did not reveal any significance in terms of demographics or psychological variables. Second, although we provided parental coaching, it was brief and the actual levels of parental understanding or execution were not assessed. Thus, parental confounding factors may not have been fully eliminated. Third, the use of IAT, a self-measured tool, as a primary outcome is a major limitation of our study and objective measurements should be utilized to overcome such limitation. Fourth, the levels of aggression and harm avoidance of the participants were not measured by biological markers such as genetic polymorphisms or functional imaging that may further elucidate the mechanism. However, regardless of the underlying mechanisms, the worse longitudinal outcome observed warrants more clinical attention.

The excessive gaming problems displayed by some children are likely to be first noticed by their parents. This may become a major parental concern because, in addition to having direct health consequences, IGD may impede necessary skill development or future career opportunities for the affected children. Parents will likely become curious about the prognosis of their already-affected children. Our findings may provide some answers, in that the gaming problems of children with high aggression and harm avoidance are less likely to resolve spontaneously. The assessment of aggression and harm avoidance levels may be clinically useful in predicting the clinical course of IGD. Active therapeutic approaches like cognitive behavior therapy should be considered in this identified risk group.

## Author contributions

S-YL conducted the analyses and drafted the manuscript. HL and SB took a part in drafting. Y-SK developed the concept and supervised the writing of the manuscript. HJ and HY further developed the concept. All authors contributed editorial comments on the manuscript.

### Conflict of interest statement

The authors declare that the research was conducted in the absence of any commercial or financial relationships that could be construed as a potential conflict of interest.
